# Bullying incidences identification within an immersive environment using HD EEG-based analysis: A Swarm Decomposition and Deep Learning approach

**DOI:** 10.1038/s41598-017-17562-0

**Published:** 2017-12-11

**Authors:** Vasileios Baltatzis, Kyriaki-Margarita Bintsi, Georgios K. Apostolidis, Leontios J. Hadjileontiadis

**Affiliations:** 1 Department of Electrical and Computer Engineering, Aristotle University of Thessaloniki, GR 54124 Thessaloniki, Greece; 2 Department of Electrical and Computer Engineering, Khalifa University of Science and Technology, P. O. BOX 127788 Abu Dhabi, UAE

## Abstract

Bullying is an everlasting phenomenon and the first, yet difficult, step towards the solution is its detection. Conventional approaches for bullying incidence identification include questionnaires, conversations and psychological tests. Here, unlike the conventional approaches, two experiments are proposed that involve visual stimuli with cases of bullying- and non-bullying- related ones, set within a 2D (simple video preview) and a Virtual Reality (VR) (immersive video preview) context. In both experimental settings, brain activity is recorded using high density (HD) (256 channels) electroencephalogram (EEG), and analyzed to identify the bullying stimuli type (bullying/non-bullying) and context (2D/VR). The proposed classification analysis uses a convolutional neural network (CNN), applying deep learning on the oscillatory modes (OCMs) embedded within the raw HD EEG data. The extraction of OCMs from the HD EEG data is achieved with swarm decomposition (SWD), which efficiently accounts for the non-stationarity and noise contamination of the raw HD EEG data. Experimental results from 17 subjects indicate that the new SWD/CNN approach achieves high discrimination accuracy (AUC = 0.987 between bullying/non-bullying stimuli type; AUC = 0.975, between bullying/non-bullying stimuli type and 2D/VR context), paving the way for better understanding of how brain’s responses could act as indicators of bullying experience within immersive environments.

## Introduction

Development of technology has enabled the connection between natural sciences and medicine with impressive results, creating the field of biotechnology^1^. A rather interesting field is the study of the human brain^2^ and particularly its electric activity^3^ during different tasks. In this field, the medical knowledge of the brain physiology is combined with the computational capabilities of modern computers and the appropriate mathematical and computational tools, in order to provide a better understanding of the human brain. Such an example is the attempt to correlate the subject’s stimulus with their electroencephalogram (EEG) recordings. In this scenario, the subject is exposed to different kinds of stimuli, which are usually pictures^4^ or sounds^5^ or both of them^6^. At the same time, the subject’s EEG activity is being recorded with the purpose of identifying the stimulus that the subject was exposed to from the EEG recordings.

In line with the aforementioned, in this research work, bullying-related incidences are attempted to be identified, based on the analysis of EEG recordings from subjects that experience bullying- and non-bullying-related visual stimuli. Conventional identification of bullying by psychologists and experts includes the use of questionnaires, conversations and tests^7,8^ that are not so realistic, as they are not synchronized with the time that the bullying events are taking place, but are rather used at a posterior moment, in which the emotional effects of the incident have worn off. Consequently, these approaches fail to reproduce the environment in which the event took place and, therefore, the results that occur from such research are not always so conclusive. With the development of technology, many different techniques of detecting school bullying have been developed, such as speech emotion recognition, mental stress recognition, and activity recognition^9,10^. Moreover, there is relative research around the study of EEG signals during relaxation and mental stress condition^11^, along with EEG-based emotion recognition^12^. However, our research work does not aim in emotion recognition, but in identifying which stimuli correspond to bullying-related incidences and which correspond to non-bullying ones in the human brain, using HD EEG signal analysis with advanced signal processing techniques. This approach is motivated by the fact that bullying is closely related with brain activity and many experts^13^ consider that the solution will come by studying the brain responses during the phenomenon. An important factor in this theory is the mirror neurons, which are a category of neurons that are activated when a person is executing an action or when they observe somebody else executing the same action^14^. Consequently, the neuron “mirrors” the behavior of the other, as thought it was acting itself. Mirror neurons have been associated with empathy, as multiple studies^15,16^ have shown activation of the mirror neuron system in empathic experiments. The low levels of empathy, which is described as the ability of a person to understand what other people feel and to put themselves in their place, in teenagers and children are amongst the strongest predictors of the phenomenon of bullying^17,18^. Therefore, there seems to be a correlation between bullying and human brain activity. However, the stagnant techniques that are being used cannot activate the brain mirror neuron system. To our knowledge, this is the first study in which an immersive environment, comprised of two-dimensional (2D) visual stimuli and virtual reality (VR), was created with the purpose of recreating realistic scenes which will provoke brain activity that is very similar to the one appearing during an actual bullying incident. VR has even been described as an “empathy machine”^19,20^ because it puts the subjects right in the middle of the action and gives them the ability to interact with their environment, thus, creating a vivid experience that leads to an even stronger coupling of the subject with the stimulus than ordinary two-dimensional visual stimuli.

The main aim of the present work is the use of an innovative combination of methods for experiential stimulation of the human brain, as well as the advanced analysis of the simultaneously recorded EEG signals, in order to efficiently identify which stimuli correspond to bullying-related incidences and which correspond to non-bullying ones. In addition to this, further distinction between 2D and VR stimuli in the bullying/non-bullying classes is addressed; moreover, gender analysis is also considered.

**Figure 1:**
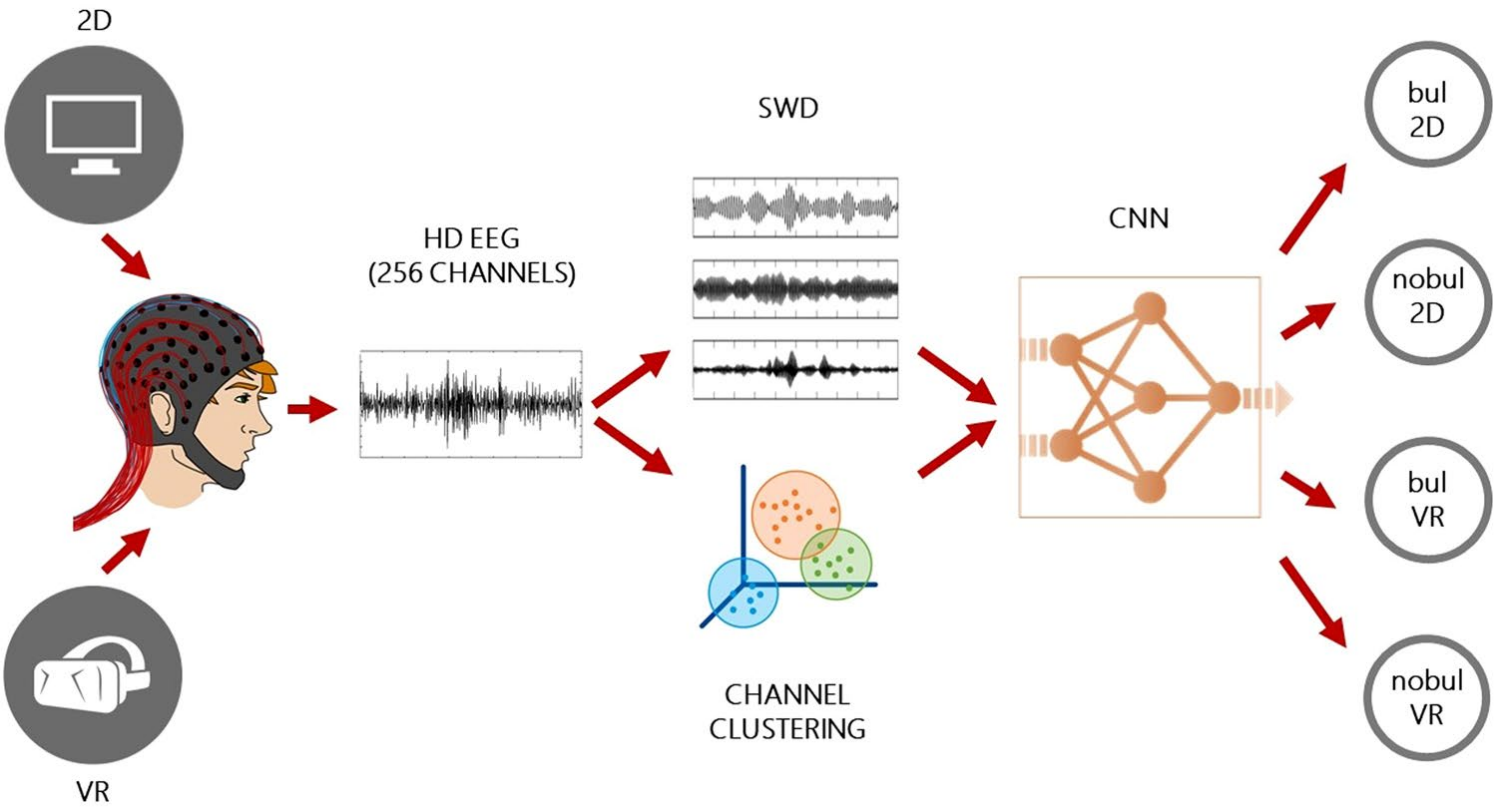
A block diagram of the proposed analysis that begins with the different kinds of stimulation (2D/VR) and the recording of the subject’s brain activity with HD EEG. The HD EEG data are then processed with SWD and the 256 HD EEG channels are spatially clustered into groups creating an image-like format. The latter are fed to a CNN, which performs a classification task in order to identify bullying (bul) and non-bullying (nobul) incidences and distinguish between the 2D and VR stimulation methods.

In order to capture the brain activity during the experiments, high-density (HD) (256 channels) EEG recordings were employed. This ensures that all of the underlying brain activity is recorded with great precision and information is not lost^21,22^. As far as the computational aspect of the work is concerned, the possibility of using deep learning algorithms^23^, and specifically convolutional neural networks (CNNs)^24^, instead of the usual machine learning algorithms^25^ was investigated for the targeted classification. Moreover, the swarm decomposition (SWD)^26^, a newly innovative approach for the decomposition of non-stationary signals based on swarm intelligence (SI)^27–29^ was also employed, in order to decompose the EEG signals into oscillatory modes (OCMs) with characteristic oscillatory pattern. Clustering algorithms^30^, like *k*-means and agglomerative hierarchical clustering, were used for the reformation of the channels, so to consider brain’s spatial information. The combination of SWD and clustering algorithms leads to the transformation of the HD EEG data to an image-like format that can be used as input to the CNN. This sequence of methods that is used here has not been applied again in the relevant literature. The procedure of the proposed analysis (all steps) is shown in the block diagram of Fig. 1.

Before arriving to the proposed analysis of Fig. 1, different combinations of the analysis steps were examined and evaluated via their classification outcome. This resulted in the following examined scenarios: {Scn1: Without SWD-Without channel clustering}, {Scn2: Without SWD-With channel clustering}, {Scn3: With SWD-Without channel clustering}, and {Scn4: With SWD-With channel clustering} (finally adopted). The CNN structures that were used included up to three times the following sequence of layers: convolutional, max-pooling, and fully-connected with logistic regression for classification. This rule was strictly followed, as more repetitions of this sequence of layers would significantly increase the CNN computational complexity and the time needed for its training. Initially, the sample was divided into 75% for training and validation set and 25% for test set. The training of the network was performed using 10-fold cross validation, where in each iteration 90% of the training sample was used for training set, and 10% for validation set. Then, the observations of the control sample were classified based on the model that occurred from the training sample.

## Results

### Gender analysis results

Prior to any further analysis, a gender analysis took place. In this study, 17 healthy subjects participated, split into 10 male and seven female (see Participants in Methods section). Considering the number of the subjects, the Mann-Whitney U non-parametric test was selected to identify statistically significant differences due to gender. More specifically, gender-related differences of the average EEG spectral power over the channels that belong to each of the 19 groups derived after spatial clustering (see Supplementary Figure S2) were tested. This analysis did not deliver any statistically significant results (*p* > 0.05); hence, the HD EEG data were grouped without taking into account gender differences.

### Scn1: Without SWD-Without channel clustering

For the case of Scn1, the CNN training was not successful and the predictions it made were equivalent of random selection. Specifically, the accuracy was 0.5043 on the validation test and 0.5079 on the test set, while the area under curve (AUC) was 0.5. This problem could be possibly solved with the use of a more complex CNN, that would include more layers, which would be able to distinguish more elaborated patterns. However, this would render the training time and computational cost infeasible.

### Scn2: Without SWD-With channel clustering

In this case, the adopted clustering of channels into groups that have spatial proximity was not able to provide a successful training of the CNN. The accuracy that was achieved was 0.4453 on the validation test and 0.4882 on the test set, while the AUC remained at a level of 0.5. Therefore, the CNN predictions in Scn2 remain equivalent of a random selection.

### Scn3: With SWD-Without channel clustering

In the case of Scn3, the CNN was successfully trained and, actually, achieved a promising level of accuracy at 0.8 on the validation set. However, a drop on the classification performance was observed on the test set, resulting in 0.5039 accuracy, 0.557 precision, 0.549 recall, and AUC equal to 0.5307. It is evident, hence, that in Scn3, over-fitting occurred and the network adapted well on the training set, yet failed to generalize so to make predictions for the instances that appear in the test set.

### Scn4: With SWD-With channel clustering

In this scenario, a significant performance was observed on all of the metrics. More specifically, the accuracy on the validation test was 0.9844 and the resulted accuracy on the test set was 0.937, the precision 0.9403, the recall 0.9395 and the AUC 0.9869. This efficient performance is also reflected in the confusion matrix^31,32^ tabulated in Table 1.

**Table 1:** Confusion matrix for the two-class problem.

		Predicted Class		Total
		Bul	NoBul	
Actual Class	Bul	47.2	5.9	53.1
	NoBul	0.4	46.5	46.9
	Total	47.6	52.4	100

The classes are Bul and NoBul. The values presented are percentages (%) of the test set and the whole test set comprised of 254 instances, 121 of which belonging to Bul class and 133 belonging to NoBul class. The corresponding classification metrics for the test set are: accuracy = 0.937, precision = 0.9403, recall = 0.9395, AUC = 0.9869.

### Four-class problem results

As it was already shown from the two-class case, the Scn4 has resulted in the most accurate results amongst the four analysis scenarios; hence, it was also adopted in the further examined four-class problem. In the latter, there was a distinction in the HD EGG data that were produced from bullying-related stimuli (Bul) and no bullying-related ones (NoBul), as well as HD EEG data that were produced from 2D stimuli (experiment T1) and VR stimuli (experiment T2), accordingly. This resulted in four combinations/classes, namely {Bul2D, NoBul2D, BulVR, NoBulVR}.

The resulted confusion matrix for the four-class problem is presented in Table 2, with corresponding test set classification metrics of 0.8858 accuracy, 0.8775 precision, 0.87475 recall and 0.975 AUC (the accuracy for the validation set was 0.9398). In order to produce the AUC for the 4-class problem, it is treated as a binary classification problem that considers one vs all scenarios. The AUC is calculated for each class and then the 4 AUCs are averaged, so that the 4-class AUC occurs. Similarly to the Scn4, high level metrics were derived, showing a very good performance even in the four-class problem. A slight drop in the classification metrics was noticed compared to the Scn4 ones, which, however, is expected, since the four-class problem adds complexity, when compared to the two-class one. Nevertheless, an accuracy level of 0.8819 on the test set in a four-class problem is considered quite satisfactory. Furthermore, if we split the problem into two complementary 2-class problems, one for 2D and one for VR, we get classification accuracy levels of 0.8055 and 0.9452, respectively.

**Table 2:** Confusion matrix for the four-class problem.

		Predicted Class				Total
		BuL2D	NoBul2D	BulVR	NoBulVR	
Actual Class	Bul2D	16.9	4.7	0	0	21.6
	NoBul2D	3.5	17.3	0	0	20.8
	BulVR	0	0	30.7	2	32.7
	NoBulVR	0	0	1.2	23.6	24.8
	Total	20.4	22	31.9	25.6	100

The classes are Bul2D, NoBul2D, BulVR and NoBulVR. The values presented are percentages (%) of the test set and the whole test set comprised of 254 instances, 52 of which belonging to Bul2D class, 56 belonging to NoBul2D class, 81 belonging to BulVR class and 65 belonging to NoBulVR class. The corresponding classification metrics for the test set are: accuracy = 0.8858, precision = 0.8775, recall = 0.87475, AUC = 0.975.

## Discussion

This is the first research study that targets the very complex problem of bullying through a novel approach, regarding not only the types of stimuli but also the means that were used to monitor the subjects’ behavior and their reaction to the stimuli. The combination of advanced signal processing, through SWD, and data mining and deep learning, through clustering and CNNs, respectively, has been proved to be quite interesting, providing efficient classification performances, both for two- and four-class problems. This confirms the initial assumptions that there is useful and substantial behavioral information at raw HD EEG waveforms and their SWD-based OCMs representations and that the use of a CNN could be able to recognize the underlying patterns and reveal such information. The results indicate that the subjects exhibited different brain activity when they witnessed bullying-related scenes and no bullying-related ones, probably affected by the triggering of their mirror neuron system in the case of bullying stimuli, due to increased empathy level. This was reflected in the interviews of the participants, from where it was deduced that all subjects distinguished between bullying and non-bullying pre-verified stimuli, since all target stimuli were correctly verified by them during the interview. Apparently, a variation in the effective empathy level existed across the subjects; yet, this was accommodated by the power of the signal processing approach, by combining SWD with deep learning. Meanwhile, the hypothesis that the immersive environment would offer a much more realistic and vivid experience than the simple 2D video was validated. The latter was confirmed by all participants via a short interview that followed the experiment (see Experimental protocol in Methods section) and was reported to be increased when moving from the experience of 2D to VR stimuli. This was potentially reflected in the derived results, as, although there is a difference in the number of incidences involved, an increasing tendency is notable in the estimated classification accuracy for the case of VR, accompanied with a corresponding lower misclassification percentage, when compared to the 2D case. Apparently, the engagement of a VR environment can deliver far more promising results than the outdated solutions that are being used so far. In fact, the proposed approach could be the start of whole new set of possibilities in the attempts to tackle bullying and could revolutionize the way psychologists approach the problem. The AMANDA project (amandaproject.net) is a venture that is moving towards this goal, which proposes a VR-based empathy museum, where the users encounter interactive bullying-related material, while their biometrics (heart-rate, RR-interval, Skin conductance) are being recorded and correlated with the stimuli. The implementation of EEG could further enhance the accuracy that the rest of the biometrics offer and could offer psychologists even better insight. This way, they could be able to provide much more targeted and specialized assistance to their bullying-affected patients.

As it has been shown from the results, the use of SWD for the decomposition of each electrode’s signal into OCMs was proved to be of crucial importance, as networks that used as an input non-decomposed data were not successfully trained. This could be attributed to the fact that the useful information was encoded in a complex way in the initial HD EEG waveforms. SWD produced simpler OCMs in a fast way, in which pattern recognition was much easier. This does not mean that there are no CNNs that could detect the existence of corresponding patterns in non-decomposed raw waveforms. However, this would require much complex networks with more levels and nodes, with a logical consequence of this being the higher demand on computing resources and longer training time.

Moreover, the use of clustering algorithms was also of equal importance for organizing HD EEG channels in groups based on topology 10–20^33^ and the distances between them. In particular, without the clustering of the channels, the problem of over-fitting emerged. In other words, the network trained very well in the data it received as an input for training but was unable to generalize its predictions for instances that differed from the training data. This may be due to the fact that convolutional filters on the network were applied to a number of channels that did not have spatial correlation with each other. However, the network practically searched for patterns in these channels considering the existence of this spatial information, so it resulted in finding patterns that were formed between non-adjacent channels, which did not record information of similar brain regions. Conversely, when the channels are grouped, the result of the convolutions with the filters includes encoded spatial information and the patterns that appear now make sense. Among the algorithms used, *k*-means produced stronger and more consistent groupings compared to the hierarchical clustering algorithm, as the resulting groups have had stronger relationships between members and weaker among them, leading to better clustering results.

The results achieved in the case of the use of SWD and grouping of channels (Scn4) were particularly satisfactory, both for the problem of the two and four classes. The high classification performance achieved is an indicator of the efficiency of the proposed approach in dealing with complex brain responses when experiencing bullying incidents within an immersive environment. To our knowledge, there is no other comparative approach, as the work described here is the first attempt that tackles such a problem, showing a good ability to identify bullying-related stimuli, shedding light upon the efficient use of brain responses for their identification.

A possible limitation of the proposed work refers to the subjects’ age, as they were University students of 21–24 years old and not teenagers, an age group in which bullying incidents mostly occur. Hence, it would be interesting to compare the findings from an experiment with subjects from this age group, with our current results and identify any differences and/or similarities due to group characteristics. Moreover, the limited number of subjects participated in the experiment sets an additional limitation; however, the breadth of the brain activity captured from each subject and the robustness of the novel approach proposed here, successfully balance such limitation. Furthermore, in this study, a regular computer screen was used for the 2D scenario and a mobile phone screen embedded within a VR headset for the VR one. Although these are distinct stimuli scenarios defined from the technical means involved in this study, some alternatives could also be explored, such as the use of wider 2D projection screens, 3D projection screens combined with 3D glasses, or mixed reality headsets (such as Microsoft Hololens). Probing further to the future, application of our approach to larger subject groups coming from different sociocultural backgrounds is foreseen, including not only controls, but pre-validated cases with different bullying experiences, combining the AMANDA concept of the gamified empathy museum with the simultaneous brain activity monitoring and psychologists’ evaluation.

## Methods

### HD EEG recording device

In each of the experiments, the brain activity was recorded using the Clinical Geodesic EEG System 400 (http://www.egi.com/clinical-division/clinical-division-clinical-products/ges-400-series), which allows the simultaneous recording of 256 EEG channels (Supplementary Fig. S1) at a sampling frequency of 250 Hz. The topology of the channels follows the 10–20 system^33^ and the Cz was used as the reference electrode.

### Participants

18 healthy subjects (11 male and 7 female) aged 21–24 years old participated in the experimental process. For the case of subject #8, the signals that were recorded had either a value of zero or some other constant value for long time periods, which corresponded to artifacts from movement and channel mis-fitting of the HD EEG cap; hence, no significant part of the recordings contained any usable information. Therefore, this subject was removed from the continuation of the survey, and, as a result, the number of participants was reduced to 17. Their participation was voluntary and anonymous, while retaining the right to withdraw from the experimental process at any time. Informed consent was obtained from all subjects involved in the study.

### Experimental protocol

In the context of the experimental protocol, two types of visual stimulation were used, namely 2D and VR. For 2D video visual stimulation (experiment T1), videos created by “The Smile of the Child” and by the 47 pupils of the High School in Pafos were used (https://www.youtube.com/watch?v=vF5OqFpp2Yw, https://www.youtube.com/watch?v=uMIdjgCE18o, https://www.youtube.com/watch?v=2LNLpgaHsWM, https://www.youtube.com/watch?v=YC61XD9LV3E). For the VR stimulation (experiment T2), videos designed and created at the Arsakeio Lyceum of Thessaloniki for the purpose of this experiment were used. The sound from all the visual stimulations was removed, with the aim that the emotional response of the subjects arises only from the visual content of the stimuli and not from a possible coupling to the music. The stimulations contained bullying-related stimuli (bul), as well as non bullying-related ones (nobul), with neutral emotional content, pre-verified by two expert psychologists. Moreover, the content of the videos used was based on guided scenarios that *a priori* were focused at the maximization of the emotional effect regarding bullying incidences. In particular, in the 2D case (experiment T1), the stimulus was displayed on a computer screen. In the first 20 seconds the subjects focused their eyes on a black background. They then watched 14 bullying-related incidences, with scenes of neutral emotional content (nobul) coming in between them. In the case of VR (experiment T2), the stimulus was viewed on a mobile, using three-dimensional video viewing software and a VR headset (http://www.homido.com). The first 20 seconds the subjects focused their eyes on a black background. Then they watched 15 bullying-related incidences, with scenes of neutral emotional content (nobul) coming in between them. HD EEG recordings, automatically synchronized with the visual stimuli, were acquired during the whole session, in both cases of T1 and T2. All participants, after each experiment, were interviewed regarding the level of empathy they felt during T1 and T2, in order to secure the correct identification of the target stimulus, i.e., no bullying/bullying stimulus. All the experimental protocols were approved by the Ethical Committee of the Aristotle University of Thessaloniki, Greece. All the experiments and recruitment were carried out in accordance with the relevant institutional guidelines.

### Data Preprocessing

The initial preprocessing included filtering at 0.3–30 Hz, artifact detection, bad channel replacement, baseline correction and segmentation, which are standard steps in EEG data preprocessing. Afterwards, a second set of preprocessing steps, which are suitable for the particular research problem, were applied. Specifically, the HD EEG data were processed, in order to have a format suitable for SWD and then to be used as input to the CNN. The initial data had the following format: 256 × 256 × 14 × 17 (channels × samples × trials × subject) for the experiment T1 and 256 × 192 × 16 × 17 (channels × samples × trials × subject) for the experiment T2.

To begin with, every signal was normalized by subtracting its mean value and dividing by its maximum value. A highpass filter at 7 Hz was then applied, so to avoid lower frequencies which could affect the speed of convergence of SWD algorithm. Subsequently, the signals were downsampled at 128 samples, from 256 and 192 for experiments T1 and T2 respectively, with the purpose of a faster SWD implementation.

### SWD

**Figure 2:**
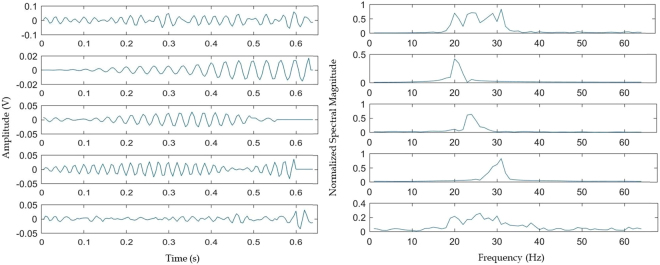
The first waveform is the initial signal, while the next three are the OCMs that the initial signal was decomposed in and the fifth waveform is the residual.

After data preprocessing, each signal was decomposed with SWD. The following parameters were used for SWD: Pth = 0.2, StDth = 0.125, Welch_nfft = 64, Welch_window = 64, Welch_no_overlap = 32. For every signal, a number of OCMs were produced. The first three OCMs were kept, while the rest were discarded, as they did not convey useful information. This way, at this point, the data had the following format: 256 × 128 × 3 × i (channels × samples × OCMs × sequential index for data used as input to the CNN). An example of the application of SWD on an EEG signal, both in time and frequency domains, is pictured in Fig. 2. From the latter (Fig. 2(b)), the localization in the frequency domain of each OCM is obvious.

### Channel Clustering

CNNs are usually used for image classification tasks. Consequently, the attempt to not only transform the HD EEG Data in a format suitable for CNN input but also take advantage of CNNs benefits sets a great challenge. The target was to extract useful information from both the temporal and the spatial dimension. Images include spatial information, since adjacent pixels represent areas of the real object which are in an equivalent distance apart. For each trial, a three dimensional matrix/image with dimensions C × τ × i was assumed, where C was the channels axis, *τ* was the samples axis and i was the OCMs axis. Therefore,the convolutional filters, that have a size greater than 1 in the channels axis, would convolve with neighboring channels, thus taking into account the spatial information among the electrodes. This kind of spatial information could prove to be very useful, since a single channel may miss the activity of an underlying brain structure, which will be recognized from the convolution with the neighboring channels.

Thus, arises the problem of representing the neighboring channels in such a way that the application of the convolutional filters is in line with the anatomical reality. Each electrode can be considered as a pixel positioned relatively to the other electrodes along the length and width of the skull. The large number of electrodes (256) covers the entire extent of the skull and there is no need for zero-padding or interpolation in the pixels that do not correspond to electrodes.

The method that is used, which is based on^34^, proposes a channel clustering according to the distances between them. The clustering applied is such a way that no electrode belongs to more than one group and the sum of the distances between the electrodes belonging to the same group is minimized. In this way, a convolutional layer can be defined that integrates the spatial information of electrodes located above similar brain regions. The *k*-means algorithm was used to group the electrodes into clusters. The Euclidean distance was used, as the electrodes have coordinates and form a geometric pattern. The number of groups was *k* = 19, according to the 19 areas defined by the 10–20 system. The coordinates of the electrodes that are considered representative for each of the regions defined by the system 10–20 were used as the initial centroids. The resulting clustering can be found in Supplementary Fig. S2.

### CNN Structure

The CNN structure^35^ that was used was a convolutional layer, followed by a pooling layer and then a fully connected layer and logistic regression for classification. More specifically, the convolutional layer is defined to have 30 8 × 8 filters. This means that each filter is applied along eight channels and eight time samples. This layer is followed by a max-pooling layer that involves a 3 × 3 matrix along the time and space axes. For instance, using a window of τ = 128 time samples, an input instance would be x ∈ ${{\mathbb{R}}}^{256\times 128}$. A filter in the convolutional layer transforms x into x ∈ ${{\mathbb{R}}}^{249\times 121}$, where the time dimension has a size of (128 − 8) + 1 = 121 and the space dimension (256 − 8) + 1 = 249.

After the convolutional layer comes the max-pooling layer which is comprised of a 3 × 3 max-filter that shifts across the time and space axes with a stride of 2. Therefore, the output has (121 − 3)/2 + 1 = 60 time samples and (249 − 3)/2 + 1 = 124 space samples, thus leading to x ∈ ${{\mathbb{R}}}^{124\times 60}$.

**Figure 3:**
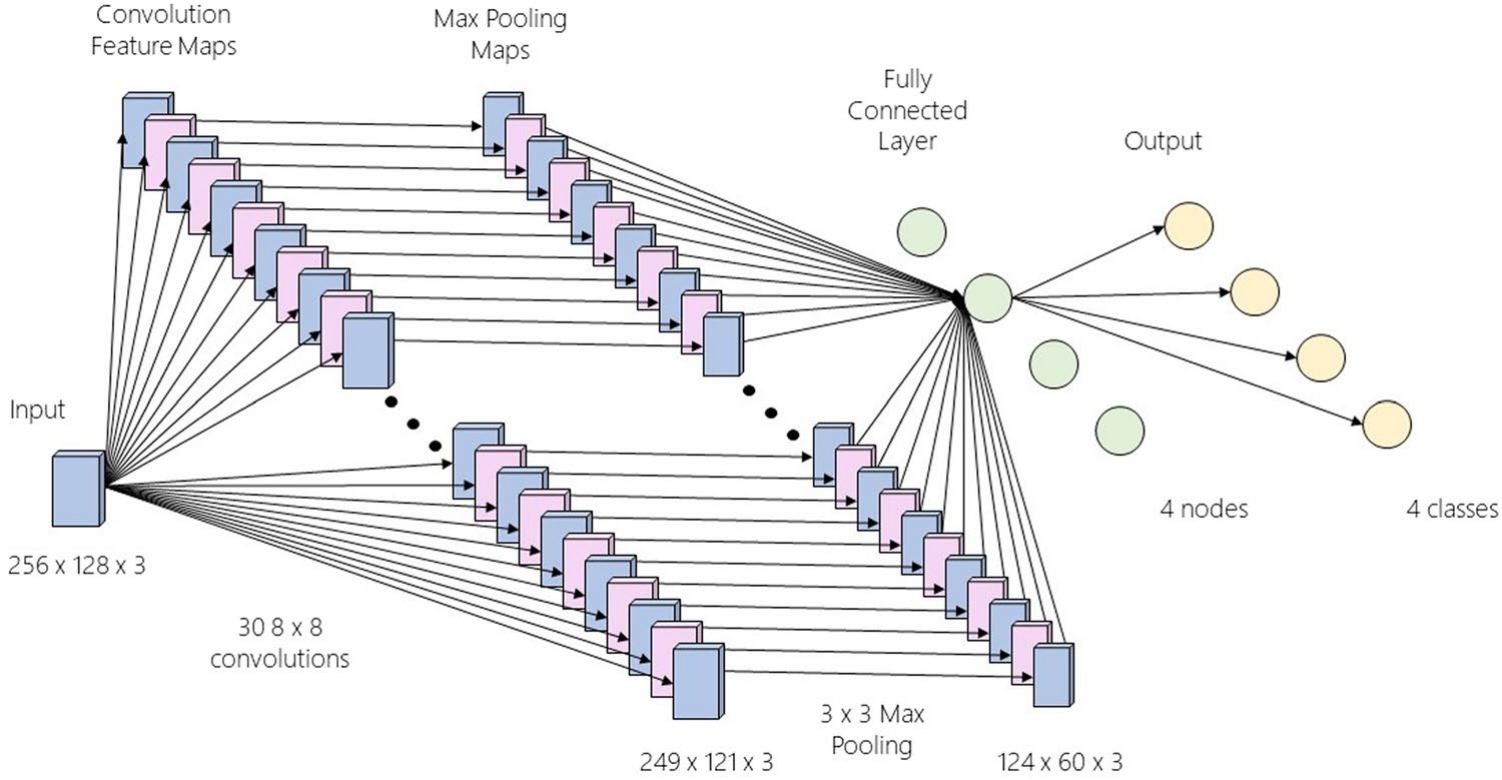
The CNN structure that was used with τ = 128. The input data have 256 channels (rows) and 128-time samples (columns). Moreover, 30 8 × 8 convolutional filters are applied on them and the resulting dimensions are 249 × 121. A max-pooling layer that involves a 3 × 3 matrix along the time and space axes is applied afterwards, resulting in new data dimensions of 124 × 60. A fully-connected layer of 2 or 4 nodes (depending on the examined problem) follows, and finally logistic regression for the classification is performed. It is important to note that all filters and functions are applied on all 3 OCMs derived from the SWD of each signal and, thus, the third dimension of all data remains unaltered through the process.

This layer is succeeded by a fully connected level of 2 or 4 nodes (depending on the problem). These nodes are used in an logistic regression (softmax) to classify the output. Every layer uses a ReLU activation function. The error function used for all outputs is defined as the cross entropy loss function. The CNN’s structure is displayed in Fig. 3.

### Data Availability

All data generated and analyzed during the current study are available from the corresponding author on a reasonable request.

## Electronic supplementary material

Supplementary Information
