# Supplementary material for: Bullying incidences identification within an immersive environment using HD EEG-based analysis: A Swarm Decomposition and Deep Learning approach
Source: Sci Rep. 2017 Dec 11;7:17292. doi: 10.1038/s41598-017-17562-0 (PMC5725430; doi:10.1038/s41598-017-17562-0)
Supplement: Supplementary file 1 — Supplementary Information [file 41598_2017_17562_MOESM1_ESM.pdf]

Supplementary Information

Bullying incidences identification within an immersive environment using HD EEG-based analysis: A Swarm Decomposition and Deep Learning approach

Vasileios Baltatzis[1, +], Kyriaki-Margarita Bintsi[1, +], Georgios K. Apostolidis[1] and Leontios J. Hadjileontiadis[1, 2, *]

[1]Department of Electrical and Computer Engineering, Aristotle University of Thessaloniki, 54124 Thessaloniki, Greece
[2]Department of Electrical and Computer Engineering, Khalifa University of Science and Technology, PO BOX 127788, Abu Dhabi, UAE
[*]leontios@auth.com
[+]These authors contributed equally to this work

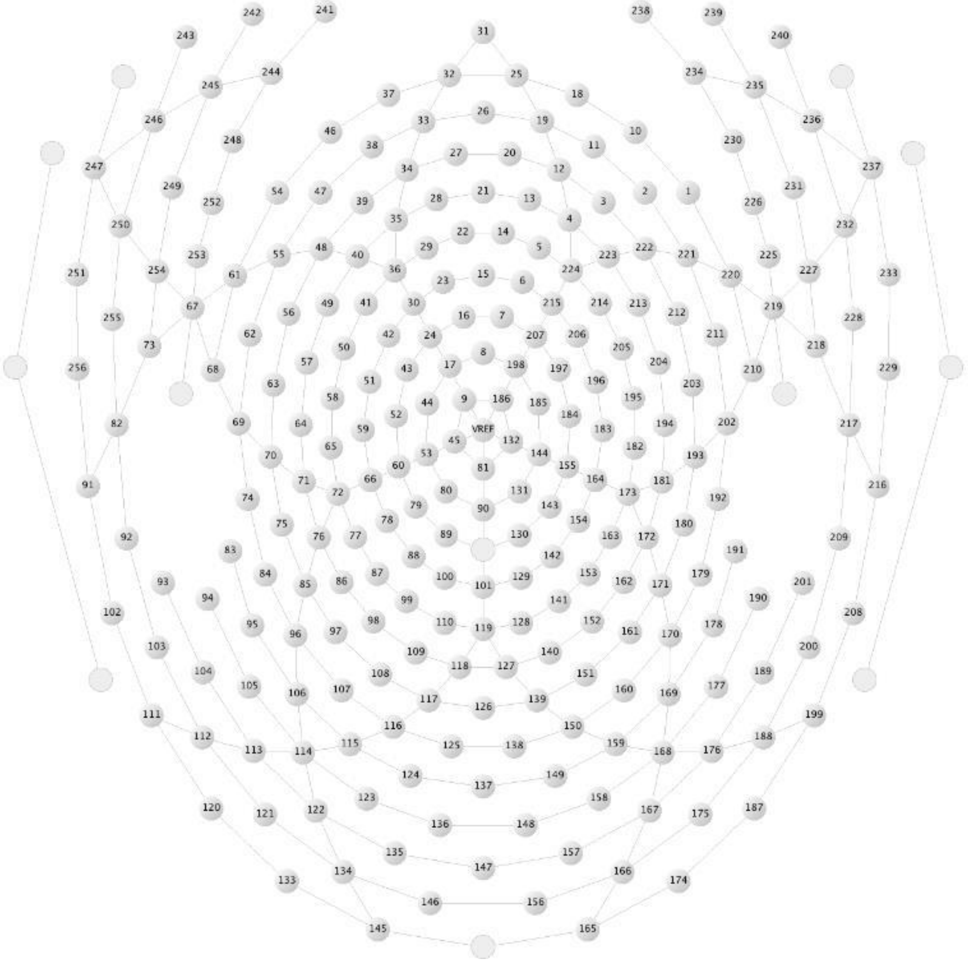

Supplementary Figure S.1. The 256 channels topology, which follows the International 10-20 system.

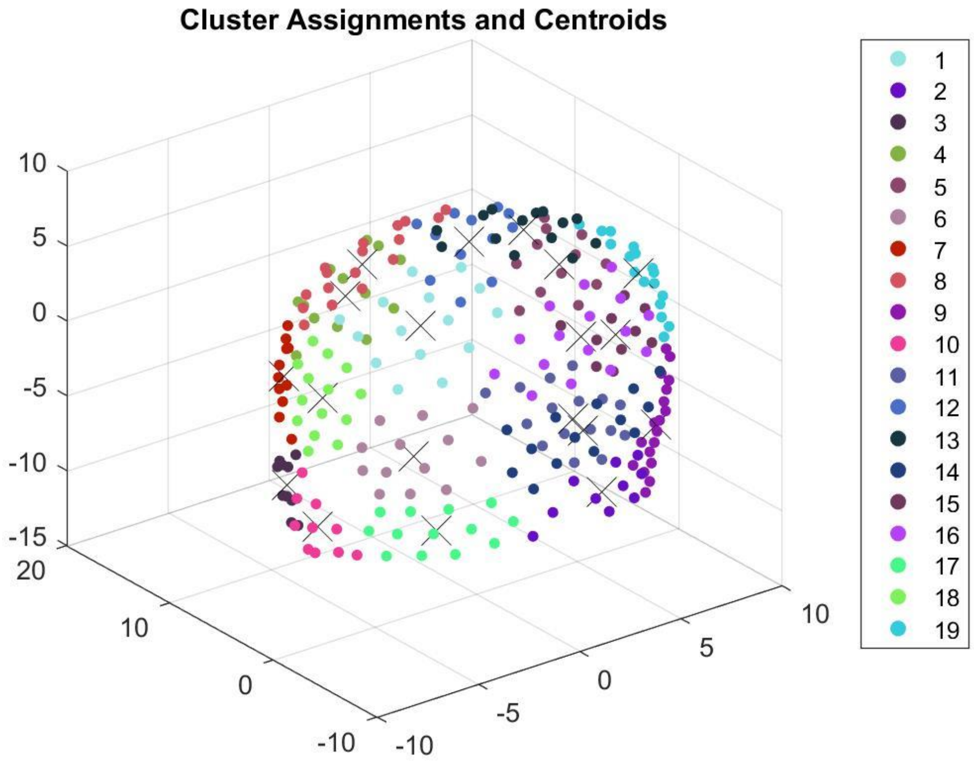

Supplementary Figure S.2. Clustering of 256 channels into 19 groups using the k-means algorithm with Euclidean distance. The members of each group are marked in different colors, while the "x" markings indicate the center of each group.
